# Kinetics of arterial carbon dioxide during veno-venous extracorporeal membrane oxygenation support in an apnoeic porcine model

**DOI:** 10.1186/s40635-015-0074-x

**Published:** 2016-01-06

**Authors:** Pedro Vitale Mendes, Marcelo Park, Alexandre Toledo Maciel, Débora Prudêncio e Silva, Natalia Friedrich, Edzangela Vasconcelos Santos Barbosa, Adriana Sayuri Hirota, Guilherme Pinto Paula Schettino, Luciano Cesar Pontes Azevedo, Eduardo Leite Vieira Costa

**Affiliations:** Research and Education Institute, Hospital Sírio-Libanês, São Paulo, Brazil; Intensive Care Unit, Hospital das Clinicas, University of São Paulo School of Medicine, Sixth floor—room 6040, Rua Dr. Eneas Carvalho de Aguiar, 255, São Paulo, 05623-010 Brazil

**Keywords:** Acute respiratory distress syndrome, Mechanical ventilation, Swine and extracorporeal membrane oxygenation

## Abstract

**Background:**

Extracorporeal membrane oxygenation (ECMO) is a technique widely used worldwide to improve gas exchange. Changes in ECMO settings affect both oxygen and carbon dioxide. The impact on oxygenation can be followed closely by continuous pulse oximeter. Conversely, carbon dioxide equilibrates much slower and is not usually monitored directly.

**Methods:**

We investigated the time to stabilization of arterial carbon dioxide partial pressure (PaCO_2_) following step changes in ECMO settings in 5 apnoeic porcine models under veno-venous ECMO support with polymethylpentene membranes. We collected sequential arterial blood gases at a pre-specified interval of 50 min using a sequence of standardized blood and sweep gas flow combinations.

**Results:**

Following the changes in ECMO parameters, the kinetics of carbon dioxide was dependent on sweep gas and ECMO blood flow. With a blood flow of 1500 mL/min, PaCO_2_ takes longer than 50 min to equilibrate following the changes in sweep gas flow. Furthermore, the sweep gas flow from 3.0 to 10.0 L/min did not significantly affect PaCO_2._ However, with a blood flow of 3500 mL/min, 50 min was enough for PaCO_2_ to reach the equilibrium and every increment of sweep gas flow (up to 10.0 L/min) resulted in additional reductions of PaCO_2_.

**Conclusions:**

Fifty minutes was enough to reach the equilibrium of PaCO_2_ after ECMO initiation or after changes in blood and sweep gas flow with an ECMO blood flow of 3500 ml/min. Longer periods may be necessary with lower ECMO blood flows.

## Background

Extracorporeal membrane oxygenation (ECMO) has been successfully used to support severely hypoxemic patients [1–7]. Despite the worldwide increase in ECMO support use, there are few studies exploring the physiology of polymethylpentene membranes in the veno-venous configuration [8, 9].

After initiation of extracorporeal support, blood flow and sweep gas flow are adjusted to achieve target values of arterial oxygen (PaO_2_), pH, and carbon dioxide (PaCO_2_) partial pressures. Titrating these ECMO settings can involve multiple steps because, frequently, the initially chosen parameters will not produce the desired result in terms of blood gases, leading to at least one more cycle of parameter change and blood gas analysis. The whole process has to be repeated whenever the patient condition changes. As a consequence of its lower solubility and lower volume of distribution throughout multiple compartments, PaO_2_ reaches equilibrium much sooner than PaCO_2_ [10]. Therefore, in order to optimize the frequency of changes in ECMO settings, it would be of utmost importance to know how long it takes for CO_2_ to equilibrate after ECMO support initiation or any parameter change.

Based on previous unpublished data, we have hypothesized that a 50-min period would be enough for PaCO_2_ equilibrium after each change on ECMO settings. Therefore, we conducted this experimental study to analyze the kinetics of arterial partial pressure of carbon dioxide following the changes in ECMO parameters during apnoeic veno-venous ECMO support.

## Methods

This manuscript is part of a sequence of experiments during porcine respiratory ECMO support, some of which were previously published elsewhere [11]. The study was approved by the Institutional Animal Research Ethics Committee from the Hospital Sírio-Libanês in São Paulo, Brazil, and was done according to the National Institutes of Health guidelines for the use of experimental animals. Instrumentation, surgical preparation, and lung injury were performed as previously described [11].

### Instrumentation and surgical preparation

Five domestic female Agroceres pigs (80 [79,81] kg) were anesthetized with thionembutal (10 mg.kg^−1^, thiopental, Abbott, Brazil) and pancuronium bromide (0.1 mg.kg^−1^, Pavulon, AKZO Nobel, Brazil) and connected to a mechanical ventilator (Evita XL Dräger, Dräger, Lübeck, Germany) with the following parameters: tidal volume 8 mL/kg, end-expiratory pressure 5 cmH_2_O, FiO_2_ initially set at 100 % and subsequently adjusted to maintain arterial saturation between 94 and 96 %, and respiratory rate titrated to maintain PaCO_2_ between 35 and 45 mmHg or an end-tidal CO_2_ (NICO, Dixtal Biomedica Ind. Com, São Paulo, Brazil) between 30 and 40 mmHg. The electrocardiogram, heart rate, oxygen saturation, and systemic pressures of the animals were monitored with a multiparametric monitor (Infinity Delta XL, Dräger, Lübeck, Germany). Central venous pressure (CVP), mean pulmonary artery pressure (PAPm), pulmonary artery occluded pressure (PAOP), and cardiac output (CO) were measured with the use of a pulmonary artery catheter. Anesthesia was maintained with midazolam (1–5 mg.kg^−1^.h^−1^) and fentanyl (5–10 mcg.kg^−1^.h^−1^), and muscular relaxation was maintained with pancuronium bromide (0.2 mg.kg^−1^.h^−1^).

**Fig. 1:**
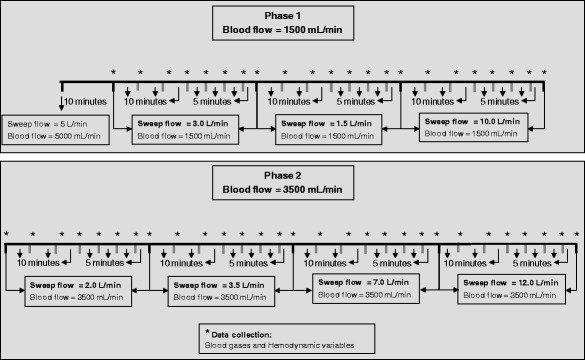
Timeline of the whole study. Blood gases and hemodynamic variables were collected after each step of an ECMO blood and sweep gas flow combination

A 25-cm ECMO arterial cannula (Edwards Lifesciences, Irvine, CA, USA) was introduced into the right external jugular vein. A 55-cm ECMO drainage cannula (Edwards Lifesciences, Irvine, CA, USA) was positioned close to the right atrium via the right femoral vein with the aid of transhepatic ultrasonographic visualization. Only the guidewires were inserted until the first baseline measurements, after the stabilization period (Fig. 1). Heparin infusion was then started at 1000 IU/h. After the first baseline, guidewires were replaced by the ECMO cannulas. Cannula diameter was chosen in accordance with vein diameter as measured with the aid of ultrasonography. Four animals had a 20-Fr and one had a 21-Fr drainage cannula. Three animals had a 21-Fr and two had a 20-Fr return cannula. A central venous catheter and an arterial line were placed in the left femoral vein and artery, respectively.

### Stabilization and support of the animals

After surgical preparation, we allowed the animals to stabilize for 1 h. A continuous infusion of 3 mL.kg^−1^.h^−1^ of lactated Ringer’s solution was infused throughout the experiment. Fluid challenges and vasopressors were used to maintain the mean arterial pressure between 65 and 80 mmHg.

### ECMO priming, starting, and maintenance

The ECMO system (Permanent Life Support (PLS) System, Jostra–Quadrox D, Maquet Cardiopulmonary, Hirrlingen, Germany) was primed with a 37 °C normal saline solution and connected to a centrifugal pump (Rotaflow, Jostra, Maquet Cardiopulmonary, Hirrlingen, Germany). Heparin infusion was titrated to keep the activated coagulation time 1.5–2.5 times its baseline value. The absence of significant re-circulation was confirmed by an oxygen saturation of less than 70 % collected from the pre-membrane port [10, 12] 10 min after the beginning of each new sweep gas flow.

### Varying ECMO blood flow and sweep gas flow

The animal was kept in apnoea with 10 cmH_2_O of PEEP and a FiO_2_ = 1.0 using a concentric coil-resistor PEEP valve (Vital Signs Inc., Totowa, NJ, USA) with a humidified oxygen continuous flow at 10.0 L/min. ECMO blood flow was initially set at 5.0 L/min, and the sweep flow was set at 5.0 L/min. After 10 min, clinical and laboratorial data were collected, and blood flow was reduced to 1500 mL/min, with an initial sweep gas flow = 3.0 L/min. An arterial blood sample was collected every 10 min for up to 30 min; afterwards, an arterial blood sample was collected every 5 min until the new ECMO adjustment was in place for 50 min. Carbon dioxide equilibrium was defined as two blood gas analysis with a PaCO_2_ variation less than 3 %. Hemodynamic data were also collected along with blood samples. After this step, the blood flow was kept at 1500 mL/min, and the sweep gas flow was set at 1.5 L/min and subsequently at 10.0 L/min in 50-min intervals. Blood flow was then elevated to 3500 mL/min, and a sequence of sweep gas flows of 2.0, 3.5, 7.0, and 12.0 L/min for 50 min each was applied (see Fig. 1). Two different blood flows were chosen in order to evaluate the impact of blood flow in PaCO_2_ equilibrium. For each ECMO blood flow, sweep gas flow sequence was chosen arbitrarily. However, for a blood flow of 1.5 L/min, we chose an alternating sequence of 3.0, 1.5, and 10 L/min in order to minimize a possible carbon dioxide carry-over phenomenon.

### Time to equilibrium–time constant (tau) determination

To calculate the time constant for each sweep gas and blood flow combination, data corresponding to the PaCO_2_ value along the time were fitted into the following equation [13]:

$$ \mathrm{Partial}\ \mathrm{pressure}\ \mathrm{of}\ {\mathrm{CO}}_2 = a + b \times \left(1-{e}^{-t/\mathrm{tau}}\right) $$

where *t* is the time in minutes, *e* corresponds to Euler’s number, *tau* is the time constant obtained for each flow combination, and *a* and *b* correspond to constants used in the model.

### Calculations

Calculations were done using standard formulas:

- Blood oxygen content C_b_O_2_ [mL O_2_/100 mL of blood] = 1.36 × Hb × Sat_b_O_2_ + 0.0031 × P_b_O_2_
- O_2_ transfer [mL/min] = (1.36 × Hb × (After - pre-membrane SatO_2_) + 0.0031 × (After - pre-membrane PO_2_)) × ECMO blood flow
- CO_2_ transfer [mL/min] = (CO_2_ partial pressure of the exhalation port of the membrane/barometric pressure) × sweep flow in mL/min
- Blood CO_2_ content [mL/min] [14] = (1 − ((0.0289 × Hb)/(3.352 − 0.456 × (Sat_b_O_2_/100) × (8.142 − pH_b_)))) × 2.226 × 0.0307 + (0.00057 × (37 − temperature)) + (0.00002 × (37 − temperature)^2^) × P_b_CO_2_ × (1 + 10 ^(pHb − 6.086)^ + (0.042 × (7.4 − pHb)) + ((38 − temperature) × 0.00472 + (0.00139 × (7.4 − pH_b_))))
- Oxygen delivery DO_2_ [mL O_2_/min] = cardiac output × CaO_2_ × 10
- Oxygen consumption VO_2_ [mL O_2_/min] = C(*a* − *v*)O_2_ × cardiac output × 10
- Standard base excess [SBE - mEq/L] = 0.9287 × (HCO3^−^ − 24.4 + 14.83 × (pH − 7.4))

### Statistical analysis

Normality was assessed by the Shapiro-Wilk test. Variables are shown as mean ± standard deviation if normally distributed and median and the 25th and 75th percentiles if otherwise. Percent variations of PaCO_2_ at the end of the 50-min observations in each sweep gas flow were normally distributed and compared with the paired *t* test. The R free source statistical package and comprehensive-R archive network (CRAN)-specific libraries were used to build the graphics and analyze the data [15].

## Results

During the entire experiment, arterial oxygen saturation (SatO_2_), pH, and standard base excess varied across the combinations of sweep gas and blood flow; however, arterial O_2_ and CO_2_ contents did not vary significantly. There were slight variations in hemoglobin and electrolytes. Table 1 shows all variables measured at the end of each 50-min period of observation

**Table 1:** Physiological variables after 50 min of sweep or blood flow modifications

	Blood flow = 1500 mL/min			Blood flow = 3500 mL/min				
Variable	Sweep flow 3.0 L/min	Sweep flow 1.5 L/min	Sweep flow 10 L/min	Sweep flow 2.0 L/min	Sweep flow 3.5 L/min	Sweep flow 7.0 L/min	Sweep flow 12 L/min	*P* value*
pH	7.21 [7.17,7.32]	7.03 [7.01,7.16]	7.26 [7.19,7.38]	7.08 [7.04,7.22]	7.16 [7.14,7.29]	7.31 [7.27,7.40]	7.43 [7.37,7.49]	<0.001
SBE (mmol/L)	0.9 [0.0,1.4]	−5.2 [−9.1,−1.2]	0.3 [−2.2,2.5]	−0.3 [−1.7,0.4]	1.2 [−1.2,2.0]	4.3 [2.9,4.9]	4.8 [4.7,6.3]	<0.001
SatO_2_ (%)	87 [81,98]	85 [75,96]	97 [80,99]	99 [91,100]	99 [91,100]	99 [96,99]	99 [98,100]	0.003
CaO_2_ (mL O_2_/100 mL of blood)	17 [14,17]	16 [13,18]	17 [14,18]	15 [14,18]	17 [15,18]	15 [15,19]	15 [14,18]	0.764
CaCO_2_ (mL CO_2_/100 mL of blood)	58 [57,64]	65 [54,66]	64 [58,66]	67 [63,74]	64 [61,64]	65 [63,65]	63 [63,69]	0.179
Lactate (mmol/L)	1.6 [1.3,2.2]	2.7 [1.7,3.6]	1.9 [1.7,4.3]	2.6 [1.1,3.4]	1.6 [1.6,3.2]	1.0 [1.0,1.8]	1.7 [0.8,2.1]	0.031
Hemoglobin (g/dL)	13 [12,14]	14 [13,14]	13 [12,13]	12 [11,13]	12 [12,13]	12 [11,13]	11 [10,13]	0.003
Chloride (mmol/L)	104 [103,104]	103 [103,104]	103 [103,104]	103 [103,104]	103 [103,104]	103 [102,105]	103 [103,104]	0.880
Sodium (mmol/L)	140 [138,143]	140 [139,142]	140 [137,141]	141 [138,141]	139 [138,142]	137 [137,139]	139 [136,139]	0.007
Calcium (mmol/L)	1.4 [1.4,1.4]	1.4 [1.3,1.4]	1.3 [1.3,1.3]	1.4 [1.3,1.4]	1.3 [1.3,1.4]	1.3 [1.3,1.4]	1.3 [1.3,1.3]	0.007
Glucose (mg/dL)	112 [103,131]	129 [116,214]	135 [115,158]	137 [124,138]	116 [112,127]	126 [121,128]	119 [118,121]	0.007
Pulmonary shunt (%)	47 [26,54]	60 [33,67]	45 [29,66]	67 [55,72]	54 [47,55]	56 [31,59]	50 [44,56]	0.115
Temperature (°C)	37.2 [36.8,38.5]	37.5 [36.6,38.4]	37.5 [36.4,38.2]	38.0 [37.0,38.1]	37.7 [36.9,38.2]	37.5 [37.0,38.2]	37.5 [37.0,38.2]	0.534
O_2_ transfer (mL/min)	116 [108,134]	115 [103,128]	133 [98,190]	246 [211,246]	201 [197,222]	206 [200,217]	207 [181,237]	<0.001
CO_2_ transfer (mL/min)	209 [204,283]	185 [178,204]	319 [275,377]	255 [223,275]	259 [238,320]	365 [294,386]	383 [296,400]	<0.001
Rotations (RPM)	2075 [1930,2175]	2075 [1980,2175]	2075 [1980,2175]	3485 [3380,3780]	3485 [3380,3780]	3485 [3380,3725]	3485 [3380,3725]	<0.001
CO (L/min)	6.5 [6.4,6.9]	6.3 [4.0,6.7]	7.7 [3.6,9.4]	7.3 [4.3,9.3]	9.0 [4.4,9.1]	7.6 [4.8,8.2]	6.3 [4.8,7.9]	0.834
Heart rate (bpm)	134 [124,145]	133 [95,137]	135 [123,147]	128 [124,149]	137 [131,160]	121 [121,153]	128 [123,161]	0.867
ABPm (mmHg)	126 [106,126]	106 [100,125]	101 [91,112]	98 [92,105]	103 [94,114]	113 [112,122]	122 [119,123]	0.154
PAPm (mmHg)	54 [41,59]	46 [42,47]	36 [32,46]	47 [34,48]	40 [31,47]	42 [35,45]	39 [33,44]	0.248
CVP (mmHg)	6 [5,13]	6 [4,16]	5 [4,13]	5 [3,15]	5 [4,14]	5 [4,11]	5 [4,11]	0.299
PAOP (mmHg)	11 [10,12]	8 [8,11]	8 [7,11]	7 [5,9]	8 [7,8]	8 [6,16]	8 [7,17]	0.715

Post hoc analyses were not done due to the high spectrum of comparisons (varying through the blood flow and sweep gas flows domains)

*SBE* standard base excess, *CaO*_*2*_ arterial content of oxygen, *CaCO*_*2*_ arterial content of CO_2_, *CO* cardiac output, *ABPm* mean arterial blood pressure, *PAPm* mean pulmonary arterial blood pressure, *CVP* central venous pressure, *PAOP* pulmonary artery occlusion pressure

*The *P* values were obtained through the Friedman’s test

**Fig. 2:**
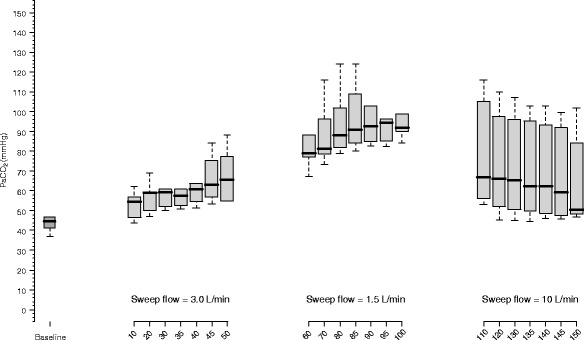
Observation of CO_2_ partial pressure during the 50-min period in each sweep gas flow step with an ECMO blood flow set at 1500 mL/min. During the baseline, the blood flow was 5000 mL/min and sweep flow was 5.0 L/min. With a fixed ECMO blood flow, lower sweep flow gas is associated with a progressive increase in PaCO_2_ values. However, a high sweep gas flow may have limited effect in lowering PaCO_2_ due to a ceiling effect with a low membrane perfusion

Variations of PaCO_2_ over time with an ECMO blood flow of 1500 mL/min are shown in Fig. 2. As expected, PaCO_2_ increased with the reduction of sweep gas flow from 3 to 1.5 L/min; however, an elevation of sweep gas flow from 1.5 to 10 L/min was associated with a slight fall of PaCO_2_ to levels similar to those observed with 3 L/min (the PaCO_2_ with sweep = 3.0 L/min was 66 [55,78] mmHg, and the PaCO_2_ with sweep = 10.0 L/min was 61 [48,92] mmHg, *P* = 0.814). In all sweep gas flows tested, the PaCO_2_ variation was not statistically different at the end of 50 min; Furthermore, a percent mean difference of PaCO_2_ between 45 and 50 min was 2 ± 3 % (*P* = 0.124, paired *t* test), −1 ± 3 % (*P* = 0.168, paired *t* test), and 4 ± 7 % (*P* = 0.338, paired *t* test) with the sweep gas flow set at 3.0, 1.5, and 10.0 L/min, respectively. However, after fitting the model of PaCO_2_ equilibrium, we observed that with a blood flow of 1500 mL/min, the time for ascendant PaCO_2_ stabilization phase had a time constant as high as 30 min, therefore an expected time to equilibrium of around 90 min. It is interesting to note that, differently from expected, after reducing the sweep gas flow from 3.0 to 1.5 L/min, the time constant fell from 33 ± 3 to 17 ± 4 min.

**Fig. 3:**
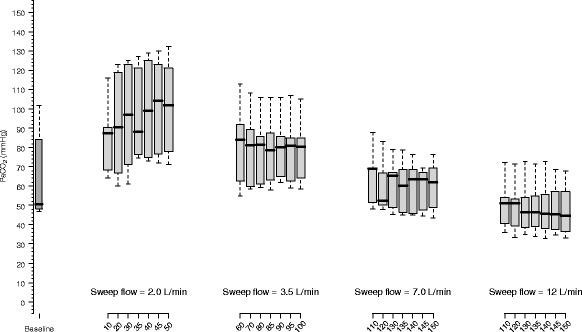
Observation of CO_2_ partial pressure during the 50-min period in each sweep gas flow step with an ECMO blood flow set at 3500 mL/min. During baseline, the blood flow was 1500 mL/min and sweep flow was 10 L/min. With a fixed ECMO blood flow of 3500 mL/min, increases in sweep gas flow are associated with a progressive decrease in PaCO_2_ values

In Fig. 3, the kinetics of PaCO_2_ with an ECMO blood flow of 3500 mL/min is shown. Unlike the previous condition, the increase of sweep gas flow up to 12 L/min was associated with a progressive reduction in the PaCO_2_. The percent mean difference of the PaCO_2_ between 45 and 50 min was 0 ± 2 % (*P* = 0.393, paired *t* test), 1 ± 1 % (*P* = 0.098, paired *t* test), −2 ± 5 % (*P* = 0.357, paired *t* test), and 2 ± 0.4 % (*P* = 0.100, paired *t* test) with the sweep gas flow set at 2.0, 3.5, 7.0, and 12.0 L/min, respectively.

**Fig. 4:**
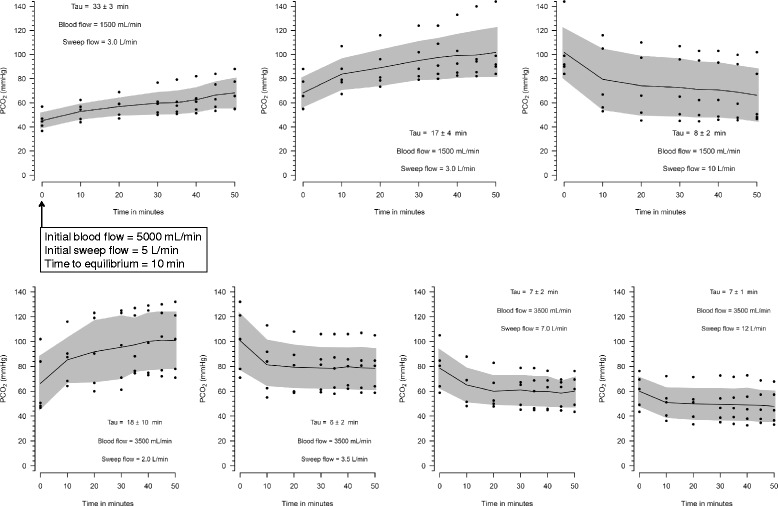
Time constant calculation for each sweep and blood flow combinations. The initial blood and sweep flow of each combination was the last combination of the precedent graphic, except for the first graph. Tau denotes time constant, and its calculation is explained in the text

Both ECMO blood flow and sweep gas flow affected the time to equilibrium of PaCO_2_ (Fig. 4). Within the studied range of sweep gas flow and blood flow, the time constant values varied from 6 to 33 min.

## Discussion

In all conditions tested, carbon dioxide had a long time to equilibrium after changes in ECMO settings. With an ECMO blood flow of 1500 mL/min, increases in sweep gas flow beyond 3.0 L/min did not significantly affect PaCO_2_. With an ECMO blood flow of 3500 mL/min, all increments of sweep gas flow up to 12 L/min resulted in additional reductions of PaCO_2_. CO_2_ equilibrium was faster with higher blood and sweep gas flows.

Unlike oxygen, which has a fast time to equilibrium and can be monitored by pulse oximetry at the bedside [16], CO_2_ requires serial arterial blood gases measurements. Differently from our initial expectative, we found long time constants for CO_2_ equilibrium with a blood flow of 1500 mL/min, suggesting that late (>50 min) assessments of arterial blood gases are usually necessary to capture the full impact of new ECMO settings in low ECMO blood flow states. Furthermore, this finding is also explained by the large volume of distribution of CO_2_ [17], also stored as bicarbonate, bound to hemoglobin, and dissolved in the peripheral tissues [14, 18, 19]. The plasma dissolved fraction (PaCO_2_) actually accounts for less than 5 % of the total body storage of CO_2_. Therefore, small variations in PaCO_2_ represent large total CO_2_ variations [20]. Because the changes in CO_2_ elimination are small in relation to its volume of distribution, the time to equilibrium is expected to be long. Moreover, after the elevation of effective ventilation, the reduction of the PaCO_2_ will depend mainly of the effective ventilation and volume of CO_2_ distribution; and after the reduction of effective ventilation, the elevation of PaCO_2_ will depend of the volume of distribution and of the CO_2_ production. The last depends on other variables such as temperature, sedation, inflammatory status, etc. Additionally, the interchange among the different molecular forms of CO_2_ depends on other modulators, such as temperature, local pH, carbonic anhydrase activity, and others [19, 21]. Thus, the time to PaCO_2_ equilibrium is a complex function derived from the combination of many variables. In this study, a 50-min period was sufficient to achieve the PaCO_2_ equilibrium in all sweep gas flow tested only with a blood flow of 3500 mL/min, otherwise in using 1500 mL/minute of blood flow, it may take as long as 90 min to reach the equilibrium.

We kept the animals apnoeic to restrict the elimination of CO_2_ to the ECMO system. With the subjects ventilating, even with a constant minute alveolar volume, changes in alveolar elimination of CO_2_ are important, because alveolar elimination depends on both alveolar CO_2_ concentration and alveolar minute ventilation [11]. We reasoned that the apnoeic model would better reflect the isolated effects of changes in ECMO settings. In Table 1, it is possible to note that CO_2_ transfer and production varied importantly between study steps and, more importantly, with an ECMO blood flow of 1500 mL/min. We attribute this finding mainly to the absence of CO_2_ stabilization throughout the phase 1 study period, instead of marked change on CO_2_ production between steps. Moreover, a variation on cardiac output and unstable animal conditions may have contributed to this finding.

At low ECMO blood flow (1500 mL/min), we found that CO_2_ elimination had a ceiling effect at sweep gas flow of 3.0 L/min with higher gas flows exerting no significant impact on PaCO_2_. This finding suggests that post-membrane CO_2_ was already very low with the sweep gas flow of 3.0 L/min, and that CO_2_ transfer was thus perfusion limited. This implies that, due to its high diffusibility, a very low CO_2_ partial pressure (its dissolved fraction) can be reached early during the passage of blood through the ECMO membrane, limiting CO_2_ elimination independently of the sweep gas flow. This finding is of great interest in situations in which ECMO is used mainly for hypercapnia or in low flow devices, such as ECCO_2_R removal technologies.

Our study has several limitations: (1) The low number of animals could lead to type-II errors, attenuating the validity of our results. However, such a limitation would not affect our positive findings. (2) We did not study animals with multi-organ failure syndrome, a condition likely closer to a clinical scenario. However, many positive associations will be intuitively the same. (3) As presented in Table 1, we observed a progressive decrease in hemoglobin concentration throughout the experiment. Considering that hemoglobin acts as a CO_2_ reservoir, this may have influenced the results. (4) We did the analysis with a static 50-min period to gas equilibrium. With low ECMO blood flows, it was not enough for PaCO_2_ to reach the equilibrium and, therefore, definitive time to equilibrium for such situation remains unknown. Moreover, it is possible that late inter-compartment distributions may occur, affecting our results.

## Conclusions

In conclusion, our results suggest that 50 min was enough to reach the equilibrium of PaCO_2_ after ECMO initiation or after changes in blood and sweep gas flow with an ECMO blood flow of 3500 mL/min. Longer periods are necessary to reach the equilibrium with an ECMO blood flow of 1500 mL/min. This may help bedside decisions and facilitate daily management of patients during ECMO support.

## Abbreviations

CO_2_: carbon dioxide
ECCO2R: extracorporeal carbon dioxide removal
ECMO: extracorporeal membrane oxygenation
O_2_: oxygen
PaCO_2_: arterial carbon dioxide partial pressure
PaO_2_: arterial oxygen partial pressure
PEEP: positive end expiratory pressure
SatO_2_: arterial oxygen saturation
